# Use of BABA and INA As Activators of a Primed State in the Common Bean (*Phaseolus vulgaris* L.)

**DOI:** 10.3389/fpls.2016.00653

**Published:** 2016-05-18

**Authors:** Keren Martínez-Aguilar, Gabriela Ramírez-Carrasco, José Luis Hernández-Chávez, Aarón Barraza, Raúl Alvarez-Venegas

**Affiliations:** ^1^Centro de Investigación y de Estudios Avanzados del IPN, Unidad IrapuatoGuanajuato, Mexico; ^2^Centro de Investigaciones Biológicas del NoroesteLa Paz, Mexico

**Keywords:** priming, common bean, epigenetics, BABA, INA

## Abstract

To survive in adverse conditions, plants have evolved complex mechanisms that “prime” their defense system to respond and adapt to stresses. Their competence to respond to such stresses fundamentally depends on its capacity to modulate the transcriptome rapidly and specifically. Thus, chromatin dynamics is a mechanism linked to transcriptional regulation and enhanced defense in plants. For example, in Arabidopsis, priming of the SA-dependent defense pathway is linked to histone lysine methylation. Such modifications could create a memory of the primary infection that is associated with an amplified gene response upon exposure to a second stress-stimulus. In addition, the priming status of a plant for induced resistance can be inherited to its offspring. However, analyses on the molecular mechanisms of generational and transgenerational priming in the common bean (*Phaseolus vulagris* L.), an economically important crop, are absent. Here, we provide evidence that resistance to *P. syringae* pv. phaseolicola infection was induced in the common bean with the synthetic priming activators BABA and INA. Resistance was assessed by evaluating symptom appearance, pathogen accumulation, changes in gene expression of defense genes, as well as changes in the H3K4me3 and H3K36me3 marks at the promoter-exon regions of defense-associated genes. We conclude that defense priming in the common bean occurred in response to BABA and INA and that these synthetic activators primed distinct genes for enhanced disease resistance. We hope that an understanding of the molecular changes leading to defense priming and pathogen resistance will provide valuable knowledge for producing disease-resistant crop varieties by exposing parental plants to priming activators, as well as to the development of novel plant protection chemicals that stimulate the plant's inherent disease resistance mechanisms.

## Introduction

As sessile organisms, plants need mechanisms to survive adverse environments, which can include pathogens. They possess structural barriers and constitutive secondary metabolites as part of their arsenal, but have also evolved diverse mechanisms of defense that “prime” their innate immune system for more robust and active induction of mechanisms against biotic and abiotic stress (Conrath, 2011). Pathogen- or microbe-associated molecular patterns(PAMPs or MAMPs), damage-associated molecular patterns (DAMPs), pathogen effectors, and wound stimuli can all initiate the defense response (Boller and Felix, 2009), which includes the priming of cells, both in tissue exposed to the inducing stimuli as well as in the systemic tissue (Conrath et al., 2002, 2006; Conrath, 2009; Jung et al., 2009). Priming can also be induced by treatment with natural or synthetic compounds, including salicylic acid (SA) and its functional synthetic analogs 2,6-dichloroisonicotinic acid (INA—the first synthetic compound to induce priming of defense responses in the lab; Kauss et al., 1992), benzothiadiazole (BTH; Katz et al., 1998), and the non-protein amino acid β-aminobutyric acid (BABA; Oostendorp et al., 2001).

Priming results in a faster and stronger induction of plant defense responses and enhanced resistance to biotic or abiotic stresses than is observed in unprimed plants exposed to the same stress (Conrath et al., 2002; Conrath, 2011). These phenomena, also referred to as “sensitization,” have been investigated for many years. As early as 1967, Kanof found that copper sulfate, a known plant irritant, caused an allergic sensitization in plants (Kanof, 1967). However, little progress on understanding the molecular bases of defense priming was achieved until the 1990s. In 1992, for example, Kauss et al. observed that pretreatment of suspension-cultured parsley (*Petroselinum crispurn*) cells with INA or SA enhanced cellular defense responses to a fungal elicitor compared to untreated cells. In more comprehensive studies, Conrath and colleagues found that pretreatment of cultured parsley cells with low concentrations of SA, INA, or BTH did not induce cellular defense responses but did induce systemic acquired resistance (SAR) in a time-dependent manner (Katz et al., 1998; Thulke and Conrath, 1998) In this same system, the effect of the inducers on expression of defense genes was assayed. Gene expression varied between genes and depended on the level of pretreatment (Thulke and Conrath, 1998; Katz et al., 1998).

Recent progress has been made in understanding the molecular bases of priming. In *Arabidopsis*, chemically induced priming is associated with the accumulation of mRNA and inactive mitogen-activated protein kinases (MPKs), specifically MPK3 and MPK6. Upon exposure to biotic or abiotic stress, these enzymes were more strongly activated in primed *Arabidopsis* plants than in non-primed plants and the activation was accompanied by enhanced expression of defense genes and development of induced resistance. Thus, accumulation of inactive MPK3 and MPK6 may be a crucial step in defense priming (Beckers et al., 2009). Furthermore, priming of the SA-dependent defense pathway is linked to histone lysine methylation, specifically, di- or tri-methylation of histone H3 at lysine 4 (H3K4me2 and H3K4me3, respectively) and to histone lysine acetylation, specifically, acetylation of histone H3 at lysine 9 (H3K9) or acetylation of histone H4 at lysine 5, 8, or 12 (H4K5, H4K8, and H4K12, respectively) at the promoter regions of the defense-associated *WRKY6, WRKY26*, and *WRKY53* genes (Jaskiewicz et al., 2011). Such modifications could create a memory of the primary infection that is associated with an amplified gene response upon exposure to a second stress-stimulus (Jaskiewicz et al., 2011). However, the connection between the action of MPKs and chromatin modifications is still unclear.

Also, it has been recently reported that the SET DOMAIN GROUP 8 (SDG8) protein, the major H3K36 di- and tri-methyltransferase in Arabidopsis (Xu et al., 2008; Li et al., 2015), is required for *Pseudomonas syringae* pv *tomato* DC3000 (PstDC3000)-triggered plant defense (Palma et al., 2010). SDG8 is required for the expression of the *R*-gene *LAZARUS 5* (*LAZ5*) and H3 lysine 36 trimethylation (H3K36me3) at *LAZ5* chromatin to maintain a transcriptionally active state. In *sdg8* mutants the H3K36me3 levels were significantly reduced at the *LAZ5* chromatin, which was associated with an absence of *LAZ5* expression (Palma et al., 2010). *LAZ5* is induced by PstDC3000 inoculation in wild-type plants but not in *sdg8* and these mutants are more susceptible to virulent and avirulent PstDC3000 (Palma et al., 2010; De-La-Peña et al., 2012). In addition, SDG8 plays a crucial role in plant defense against necrotrophic fungal pathogens through H3K36me3-mediated activation of a subset of genes within the JA/ET signaling defense pathway (Berr et al., 2010). All together, the active H3K4 and H3K36 methylation states, catalyzed by SET domain-containing proteins, have been implicated in the SA- and JA-mediated plant defense in *Arabidopsis*. These methylation states generally associated with transcribed genes act as permissive marks for the basal expression of defense genes, or by establishing the chromatin status that enables a plant to react more rapidly when challenged (Alvarez-Venegas, 2010; Ding and Wang, 2015).

The priming status of a plant can be inherited to its offspring. There are at least two reports of transgenerational priming in the progeny of plants exposed to *Pseudomonas syringae* or BABA. In *Arabidopsis*, SAR was inherited epigenetically after inoculation with *P. syringae* pv *tomato* DC3000 (PstDC3000; Luna et al., 2012). This conclusion was based on the finding that the progeny from the inoculated plants were primed for enhanced activation of the *PR1, WRKY6*, and *WRKY53* genes associated with immunity to *Hyaloperonospora arabidopsidis* and PstDC3000 when compared to progeny from non-inoculated plants. In a second study, progeny of *Arabidopsis* that had been either primed with BABA or inoculated with *P. syringae* pv *tomato* (avrRpt2) had enhanced expression of defense-related genes that confer resistance to PstDC3000 and *H. arabidopsidis* (Slaughter et al., 2012). Collectively, these two studies in *Arabidopsis* suggest the importance of epigenetic mechanisms in transgenerational priming for induced resistance.

Legume plants, particularly the common bean (*Phaseolus vulgaris* L.), are an excellent model system to study plant-pathogen interactions and priming. Common bean is an important crop worldwide and the most important grain legume in the human diet. It is affected by a number of pathogens, including pathogenic fungi, bacteria, and viruses. Thus, it is surprising that there are only a few reported studies of priming in the interaction of this important crop with its pathogens (for an example, see Siegrist et al., 1997).

In the study reported herein, resistance to *P. syringae* pv. phaseolicola infection was induced in the common bean with the synthetic priming activators BABA and INA. INA and BABA were chosen as the elicitors of priming because they have been shown to induce gene priming in *Arabidopsis* (Kessmann et al., 1994; Ton and Mauch-Mani, 2004). Resistance was assessed by evaluating symptom appearance, pathogen accumulation, changes in gene expression of defense genes, as well as changes in the H3K4me3 and H3K36me3 marks at the promoter-exon regions of defense-associated genes. We conclude that defense priming in the common bean occurred in response to BABA and INA and that these synthetic activators primed distinct genes. According to our results, INA induced transgenerational priming for enhanced resistance. Gene expression correlated with permissive histone marks.

We hope that an understanding of the molecular changes leading to defense priming and pathogen resistance will facilitate the development of engineered plants with enhanced disease resistance and to the development of novel plant protection chemicals that stimulate the plant's inherent disease resistance mechanisms.

## Materials and methods

### Plant material

Wild-type *Phaseolus vulgaris* cultivar BAT 93 seeds and plants were used in this study. Seeds were surface sterilized with 2% sodium hypochlorite, rinsed five times, placed in sterile plates, covered with tin foil, and germinated under sterile conditions for 3 days at 28°C. The seedlings were transferred to 2.3 L pots containing vermiculite and placed in a greenhouse, located in Guanajuato, México (101°09′01″ west longitude, 20°30′09″ north latitude; 1730 meters above sea level). The facility comprises 10 independently controlled beds arranged side-by-side in a 5 × 2 array, with rows of beds running east/west and columns of beds running north/south. Each bed is separated from its neighbors by a corridor, and the columns have mirror symmetry with respect to paths, ventilators, etc. Every bed accommodates three rows of 30 plants, giving 90 experimentally independent plants per bed. The experiments were from June to September 2014 and 2015, under daylight conditions (14 h light; 10 h darkness), with an average maximum and minimum temperatures of 30 and 18°C, respectively.

All plants were watered every other day and once a week they were fertilized with a B&D nutrient solution (CaCl_2_, 1 mM; KH_2_P0_4_, 0.5 mM; ferric citrate, 10 μM; MgSO_4_, 0.25 mM; K_2_SO_4_, 0.25 mM; MnSO_4_, 1 μM; H_3_BO_3_, 2 μM; ZnSO_4_, 0.5 μM; CuSO4, 0.2 μM; CoSO_4_, 0.1 μM; Na_2_MoO_4_, 0.1 μM; Broughton and Dilworth, 1971) supplemented with nitrogen (8 mM KNO_3_; Estrada-Navarrete et al., 2007).

### Bacterial strain and growth conditions

*Pseudomonas syringae* pv. phaseolicola NPS3121 (PspNPS3121) was grown on KB (King's B medium - Recipe, 2009) media containing Rifampicin (50 μg/mL).

### Priming activators

The synthetic priming activators used in this experiment were 2,6-dichloropyridine-4-carboxylic acid (also known as dichloroisonicotinic acid, INA; Sigma-Aldrich, catalog #456543-1G), and β-aminobutyric acid (BABA; Sigma-Aldrich, catalog #A44207-5G).

Foliage leaves, or first true leaves, from 10-day-old plants, seven days after germination (dag), were sprayed once with 2 mL per plant of 100 μM INA, and later on challenged with the pathogen. Care was taken to cover the apical meristem in order to avoid direct contact with the activator. In another group of plants (all growing independently in 2.3 L pots containing vermiculite), the soil was drenched with 50 mL per plant of 100 μM BABA, and later on challenged with the pathogen. Separate batches of plants were grown and then subjected to INA or BABA treatment only (without pathogen treatment). A parallel group of non-activated control plants was kept and later exposed to biotic stress to provide a thorough comparison of INA- or BABA-treated and non-induced plants. Control plants were treated in the same way, but with water. Samples were taken, in triplicate, from distal leaves that had not been directly exposed to the INA activator 24 h before and 24 h after the application of the activators. Samples from BABA-treated plants (leaves from the same developmental stage as INA-treated plants) were taken, in triplicate, 24 h before and 24 h after the application of the activator. All samples were stored at −80°C.

### Pathogen infection

The abaxial surface of two lower trifoliate leaves that had not been treated with INA were infiltrated with the pathogen 1 week after application of the priming activator (14 dag). Trifoliate leaves from BABA-treated plants, at the same developmental stage as INA-treated plants, were also infiltrated with the pathogen 1 week after application of the priming activator.

*P. syringae* were cultivated at 28°C, 220 rpm on KB media containing 50 μg/mL rifampicin for selection, centrifuged, and then were suspended in 10 mM MgCl_2_ at a final concentration of 5 × 10^8^ colony-forming units (CFU)/mL and placed in a syringe without a needle. The tip of the syringe was pressed against the underside of the leaf while simultaneously applying gentle counter-pressure to the other side of the leaf. Six infiltration points (each 1 cm^2^) per leaf were used to expand the infiltration area. Positive control plants were not infiltrated, but were treated with the priming activators. Negative control plants were neither treated nor infiltrated. Samples were taken from distal leaves that had not been exposed neither to the activators nor to the pathogen, 24 h before infection and 24 h and 120 h after infection. Samples were stored at −80°C.

An outline of the experimental design used in the presented study is given in Table 1.

**Table 1:** **Outline of the experimental design used in the presented study**.

**Activator**	**Inoculation**	**Activator**	**Inoculation**
BABA	PspNPS3121	INA	PspNPS3121
BABA	- - - - - -	INA	- - - - - -
BABA	water	INA	water
- - - - - -	PspNPS3121	- - - - - -	PspNPS3121
- - - - - -	- - - - - -	- - - - - -	- - - - - -

### Disease evaluation

Bacterial growth in the plant tissue was assessed 10 days after infection as follows: Leaf discs of area 1 cm^2^ that were contiguous to the infection sites were excised, rinsed, homogenized in sterile distilled water, and plated in serial dilutions (1:10; 1:100; 1:1000) on KB media containing 50 μg/mL rifampicin. The plates were incubated at 28°C for 36 h and the total number of CFUs from two plates for each dilution was determined. Fiji software (Schindelin et al., 2012) was used to calculate the percentage of leaf damage (total leaf area over total necrotic leaf area), on leaves that were challenged with the pathogen.

### Candidate genes with possible priming effect

We wished to identify genes in the common bean that were likely targets for priming events that confer resistance to plant pathogens. We performed a search in multiple databases as well as in the few published studies on defense priming for these genes, which might serve as markers for priming. The following common bean genes, orthologs to Arabidopsis genes, were chosen: *PvWRKY6* (*Phvul.011G101900*), *PvWRKY29* (*Phvul.002G293200*), *PvWRKY53* (*Phvul.002G296700*), *PvPR1* (*Phvul.006G196900*), *PvPR4* (*Phvul.006G102200*), and *PvNPR1* (*Phvul.006G131400*). The relevance of these genes was analyzed by the quantification of their transcripts by PCR in response to the priming activators and to the pathogen and for chromatin marks associated with active genes at their promoter regions.

### cDNA synthesis and qRT-PCR analysis

PureLink RNA Mini Kit (Life Technologies, Carlsbad, CA, USA) was used to isolate total RNA from all the samples. For quantitative real-time PCR (qRT-PCR) analysis, RNA was treated with DNaseI (Invitrogen, Carlsbad, CA, USA) to remove genomic DNA. The absence of DNA was confirmed by performing PCR on the DNaseI treated RNA using Taq-DNA polymerase. One microgram of total RNA was reverse transcribed in 20 μL reaction mix with 200 U of SuperScript II-Reverse Transcriptase (Invitrogen, Carlsbad, CA, USA) and oligo(dT) primer. A StepOne® Real-time PCR system (Applied Biosystems, Carlsbad, CA, USA) was used for real-time PCR quantifications. qRT-PCR was performed according to the Maxima® SYBR Green/ROX qPCR Master Mix (2x) protocol (Thermo Scientific, Waltham, MA, USA). A “no DNA” template control was used in each analysis. The method used to analyze the data from real-time PCR experiments corresponds to the relative quantification method, or 2^−ΔΔCT^ method, where the ΔΔCT value = ((CT_1*Target*_ – CT_1*Reference*_) – (CT_0*Target*_ – CT_0*Reference*_)) (Livak and Schmittgen, 2001). The mean CT values for both the target and internal reference genes were determined and the fold change in the target gene normalized to *PvAct11* and *PvEF1*α, and relative to the expression in the control sample. Primers used were as follows: for *PvWRKY6* (*Phvul.011G101900*), PvuWRKY6F 5′-aagccactgttagcacc aaagacaccatc-3′, and PvuWRKY6R 5′-ggatgatgg ggaagagtcatgattgtttggagaac-3′; for *PvWRKY29* (*Phvul.002G293200*), PvuWRKY29F 5′-tttgcaccctctttcacctcacaccatc-3′, and PvuWRKY29R 5′-tttgagttctttgacttgttggggttctatgggg-3′; for *PvPR1* (*Phvul.006G196900*), PvuPR1F 5′-cacaaaactcaccccaagacttcctcaa-3′, and PvuPR1R 5′-ttgcatcccatctcattggtcctacc-3′; for *PvPR4* (*Phvul.006G102200*), PvuPR4F 5′-gcagaatactgttcaccctctca-3′, and PvuPR4R 5′- gttgagcagtaagcactcacgg-3′; for *PvWRKY53* (*Phvul.002G296700*), PvuWRKY53F 5′-gggcagaaaggccctgaaggagaa-3', and PvuWRKY53R 5′-agcggtgaaattggtgtcgttg agga-3′; for *PvNPR1* (*Phvul.006G131400*), PvuNPR1F 5′-ggttgtctctgaggtgcttggt ttggg-3′, and PvuNPR1R 5′-caatatgaagcaccga gtagccccgag-3′.

The results presented are from three independent biological replicates from different plants. Each biological replicate was tested in triplicate and data were normalized to the Actin11 (*PvActin11*) reference gene (PvActin11F5′-tgcatacgttggtgatgagg-3′, and PvActin11 R5′-agccttggggttaagaggag-3′ (Borges et al., 2012), and to the elongation factor 1-α (*PvEF1*α) reference gene (PvEF1aF 5′-ggtcattggtcatgtcgactctgg-3′, and PvEF1aR 5′-gcacccaggcatacttgaatgacc-3′; Livak and Schmittgen, 2001; Barraza et al., 2013, 2015). Statistical significance for the F0 and F1 generations was determined with multiple Student's *t*-test, followed by the Holm-Šídák multiple comparison test at a significance value of 0.05, by using the GraphPad Prism (v 6.0, GraphPad Software, San Diego California USA, http://www.graphpad.com). For the F1 generation one-way ANOVA with Dunnett's post-test was performed using GraphPad Prism (v 6.0, GraphPad Software, San Diego California USA) at a significance value of 0.05.

### Chromatin immunoprecipitation

Chromatin isolation and immunoprecipitation were performed as described previously (Alvarez-Venegas and Avramova, 2005; Saleh et al., 2008), with some modifications. In brief, the chromatin was sheared by sonication to 500–1000 bp fragments, the sample was centrifuged for 10 min at 13,000 r.p.m., the sonicated chromatin was collected and was frozen at −80°C until it was immunoprecipitated.

Before immunoprecipitation, calibration curves were created to establish the best possible amounts of chromatin to be used and to ensure comparable amounts of starting material (Alvarez-Venegas and Avramova, 2005). Aliquots of the sonicated chromatin (diluted 10-fold) were used in each immunoprecipitation, for which we employed the EZ-Magna ChIP kit (Catalog #17-408, Millipore), the Magna ChIP Protein A + G Magnetic Beads (Catalog #16-663, Millipore), and followed the manufacturer's instructions. The immunoprecipitation was performed overnight at 4°C, using 5 μL of the respective antibodies. The antibodies were: anti-histone, CT, pan, Clone A3S (Catalog #04-928, Millipore), ChIPAb + Trimethyl-Histone H3 (Lys4; Catalog #17-614, Millipore), and ChIPAb + Trimethyl-Histone H3 (Lys36; Catalog #17-10032, Millipore). Following immunoprecipitation, the Protein A + G bead-antibody/chromatin complex was separated with the magnetic separator (Magna Grip Rack; Catalog #20-400, Millipore), washed, the protein-DNA complexes eluted, and crosslinks of protein/DNA complexes reversed to free the DNA. The DNA purification was performed with the Illustra GFX PCR and DNA Gel Band Purification Kit (Catalog #28-9034-66, GE Healthcare), following the manufacturer's instructions.

An aliquot of the initial sonicated chromatin, where the crosslinks of protein/DNA complexes were reversed and the sample purified, was used as template for input samples. Amplification was carried out by using Platinum TAQ High Fidelity DNA polymerase (Thermo Fisher Scientific Inc.). All PCR reactions were performed in 25 μl: one cycle of 5 min at 95°C, then 35 cycles of 95°C for 30 s, 58°C for 30ṡ, 72°C for 2 min, and followed by 72°C for 5 min. Primers used were as follows: for *PvWRKY6*, PvuWRKY6F 5′-attgccgagtccattgcatcgt-3′; and PvuWRKY6R 5′-tctgttgtggagggatgatgggg-3′; for *PvWRKY29*, PvuWRKY29F 5′-tgaactctcaccgtccaacaacca-3′; PvuWRKY29R 5′-caaggaccctggtg gtttctgaga-3′; for *PvWRKY53*, PvuWRKY53F 5′-tgcca ccatccactaaatctgccc-3′, and PvuWRKY53R 5′-gcc ttgccaactccaaaccttgta-3′; for *PvPR1*, PvuPR1F 5′-atc ccaatgcttcctttggtagcg-3′; PvuPR1R 5′-aaggttctccccataa ggaccccc-3′; for *PvPR4*, PvuPR4F 5′- gtctgaaactcaatcct ctagcagccattcttatctc-3′, and PvuPR4R 5′-cccatctctctctcac tttaaattcagaacttttcccaa-3′; for *PvNPR1*, PvuNPR1F 5′-gct ggggacgctgatgtcatctat-3′, and PvuNPR1R 5′-ctga aagggaaacaaagaagggcg-3′. PCR products were electrophoresed on 2% agarose gels. Band intensities were quantified using the Image Lab v 4.0 software (Bio-Rad Laboratories Inc.). Intensities were normalized versus the input sample, representing 15% of the DNA used as template (Alvarez-Venegas and Avramova, 2005). Each ChIP experiment was independently performed in duplicate.

### Transgenerational inheritance of priming

All common bean plants (designated the F0 generation), from all the different treatments (activator only, activator plus pathogen, and pathogen only), were self-pollinated and grown to seed set to generate the F1 progeny. These progeny were analyzed for disease resistance and expression of priming-related genes. Seeds from the F1 progeny were grown and infiltrated with *P. syringae* as described earlier with the following modifications. No priming activators were used in the F1 progeny (neither BABA nor INA). Positive control plants were not infiltrated, but treated with water. Negative control plants were not infiltrated nor treated with water (Table 2). Samples were taken, in triplicate, 24 and 120 h after the infection from distal leaves that had not been exposed to the pathogen and stored at −80°C.

**Table 2:** **Analysis of F1 progeny of *P. vulgaris* plants**.

**F0**	**F1**
BABA + PspNPS3121	PspNPS3121
No activator + PspNPS3121	PspNPS3121
INA + PspNPS3121	PspNPS3121
No activator + PspNPS3121	PspNPS3121
- - - - - -	- - - - - -

F0 plants were self-pollinated and grown to seed set to generate the F1 progeny. Seeds from the F1 progeny were grown and infiltrated with P. syringae, as shown.

## Results

### *P. vulgaris* plants treated with activators exhibit phenotypic characteristics of defense priming against *P. syringae* pv. Phaseolicola

**Figure 1:**
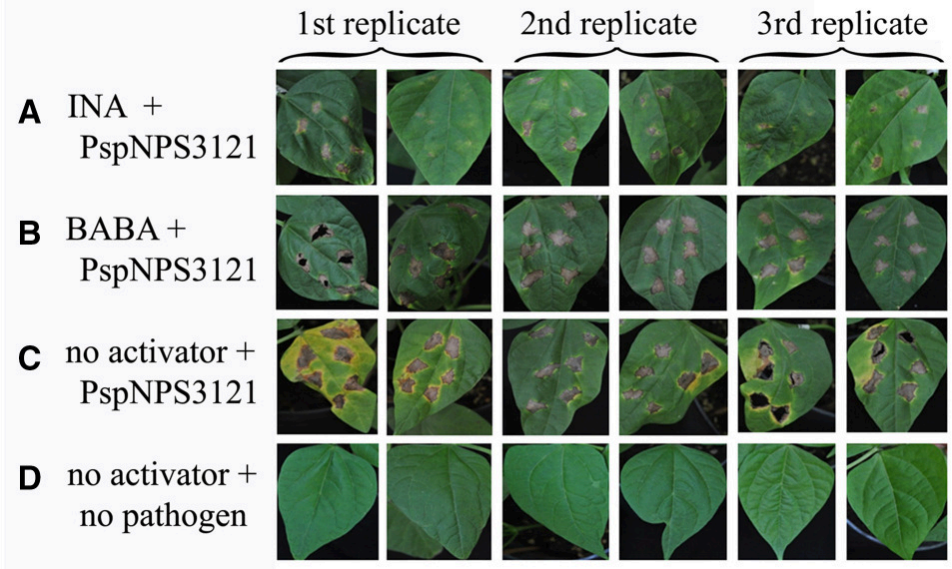
**Lesion development in leaves from *P. vulgaris* plants inoculated with *P. syringae* pv. phaseolicola NPS 3121 (PspNPS3121) after treatment with (A) INA, (B) BABA, or (C) water; (D) Non-inoculated, untreated control plants**. Photos were taken 10 days after pathogen inoculation.

Common bean plants were treated with water or one of the activators, INA or BABA, and challenged 1 week later with *P. syringae* pv. phaseolicola NPS3121 (PspNPS3121), which causes halo blight in common bean (Figure 1). This experiment was performed to determine if the activators “prime” the plants for resistance to the pathogen. As can be seen in Figure 1, application of INA or BABA before bacterial infection resulted in a robust resistance, after pathogen inoculation, against the halo blight caused by *P. syringae* (PspNPS3121). We conclude that plants treated with the synthetic priming activators were effectively protected against the pathogen, in contrast to plants that were not primed.

**Figure 2:**
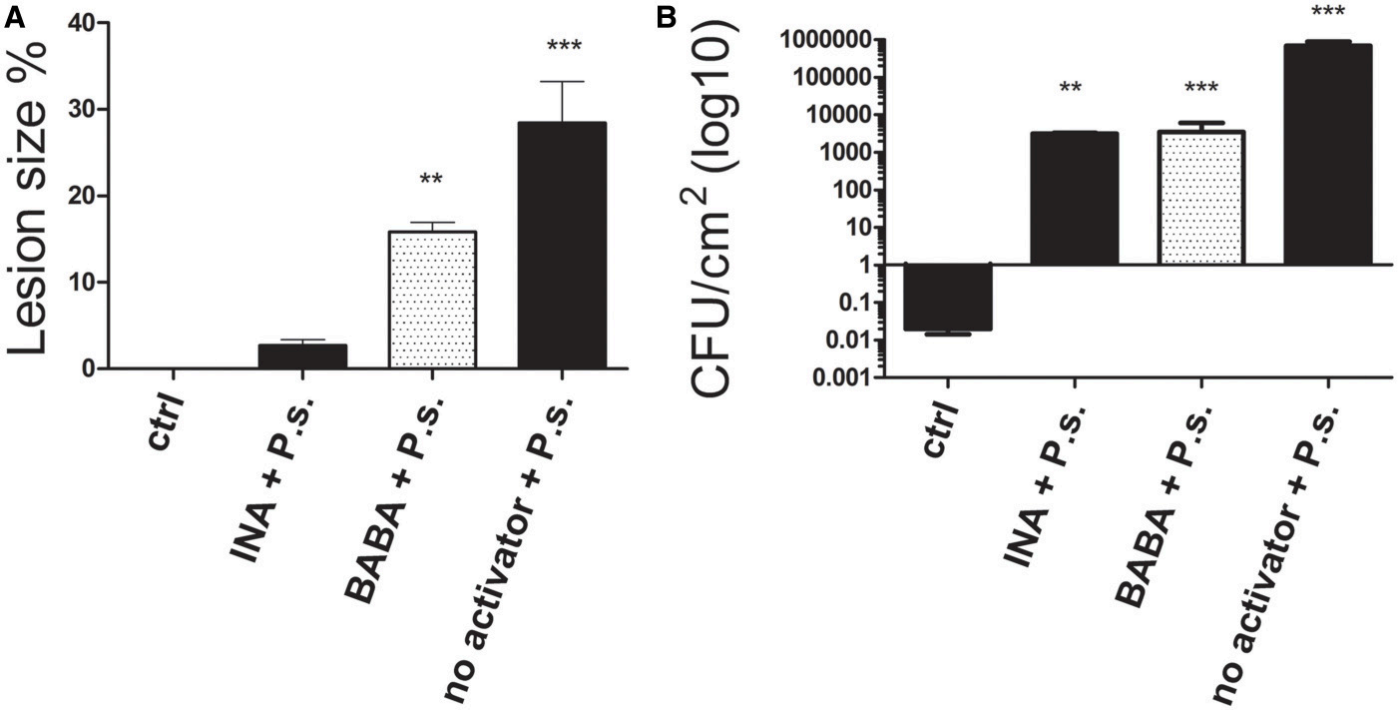
**(A)** Lesion size and **(B)** colony-forming units (CFU) of *P. vulgaris* plants 10 days after inoculation with *P. syringae* (P.s). Control (ctrl) plants were neither treated with activator nor inoculated. Data represent mean ± SD, *n* = 3 independent experiments; data were analyzed with an unpaired two-tailed Student's *t*-test (^**^p < 0.01, ^***^p < 0.001).

Treatment with the activators induced a 90.55 and 44.45 % reduction in lesion size for INA and BABA, respectively, compared to the untreated, inoculated plants 10 days after inoculation (Figure 2A). Figure 2B shows that the bacterial populations in leaves from the treated plants were lower than in the untreated, inoculated plants, which showed typical disease progression. INA treatment reduced bacterial growth by 99.35% and BABA reduced it by 99.5%, compared to the untreated, inoculated plants. These results confirm our conclusion that the priming activators protected common bean plants from the pathogen.

### INA and baba prime plants for higher expression of some genes in the common bean

The next experiments were undertaken to examine the effect of priming on the accumulation of transcripts of six genes that were identified as related to priming and possible priming targets. When transcript levels of plants (systemically resistant leaves) that had been primed with activators and inoculated with the pathogen were initially monitored by end-point PCR, *PvWRKY6, PvWRKY29, PvWRKY53, PvPR1, PvPR4*, and *PvNPR1*, clearly showed the biphasic transcript accumulation pattern that is typical of the priming response (Supplementary Figure 1). That is, the activators did not trigger the expression of the genes. However, after inoculation of the primed plants with PspNPS3121, transcripts accumulated over the levels of the unprimed, inoculated controls.

**Figure 3s:**
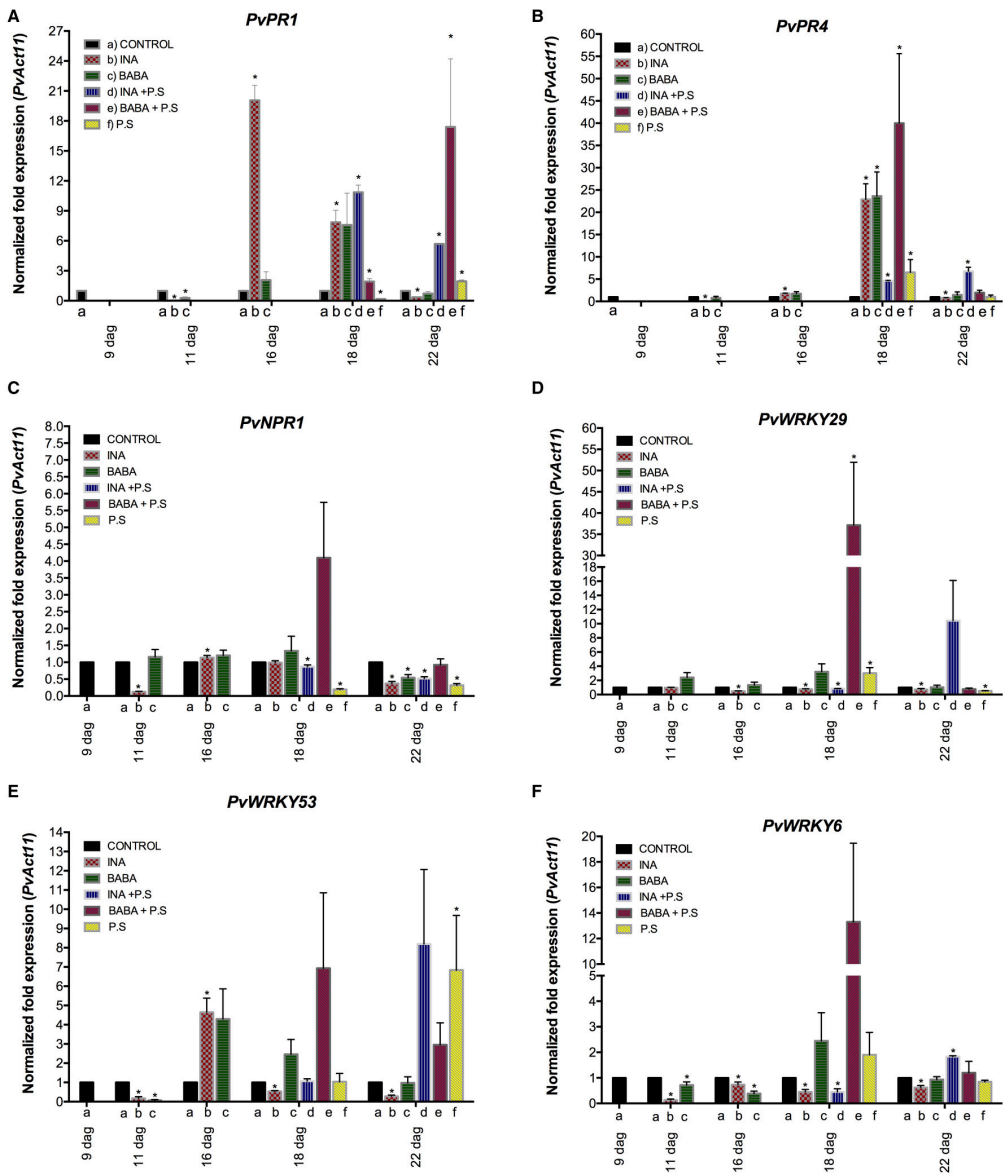
**Transcript levels of genes from *P. vulgaris* involved in plant defense as determined by qRT-PCR at various days after germination (dag)**. Plants were primed with activators (BABA- or INA-treated plants) followed by inoculation with *P. syringae* pv. phaseolicola (Activator + P.s.), inoculated only (no activator + P.s.), or neither primed nor inoculated (control, ctrl). Data were normalized to the Actin11 (*PvActin11*) reference gene. **(A)** *PvPR1*; **(B)** *PvPR4*; **(C)** *PvNPR1*; **(D)** *PvWRKY29*; **(E)** *PvWRKY53*; **(F)** *PvWRKY6*. Data represent mean ± SD, *n* = 3 independent experiments. Statistical significance for the F0 generation was determined with multiple Student's *t*-test, followed by the Holm-Šídák multiple comparison test at a significance value of 0.05 (^*^p < 0.05), by using the GraphPad Prism (v 6.0, GraphPad Software, San Diego California USA, www.graphpad.com) (see Supplementary Table 1).

We next verified and quantified gene expression more accurately with qRT-PCR. We analyzed the transcript levels of *PvWRKY6, PvWRKY29, PvWRKY53, PvPR1, PvPR4*, and *PvNPR1* in primed, inoculated plants. As shown in Figure 3 (and in Supplementary Figure 2), each of the priming activators primed distinct genes, with enhanced gene expression at different time points. BABA primed the six genes tested (*PvWRKY6, PvWRKY29, PvWRKY53, PvPR1, PvPR4*, and *PvNPR1*), and INA primed two of the six genes tested (*PvWRKY29* and *PvWRKY53*).

In systemic leaves of BABA-treated plants, priming alone did not enhance transcription of *PvWRKY6, PvWRKY29, PvWRKY53, PvPR1, PvPR4*, and *PvNPR1* (Figure 3). However, 24 h after bacterial inoculation, there was strong accumulation of *PvPR4, PvNPR1, PvWRKY29, PvWRKY53*, and *PvWRKY6* transcripts, which decreased 120 h after bacterial inoculation, in BABA-treated plants as compared to control plants (Figures 3B–F, respectively). In contrast, there was an enhanced transcription of *PvPR1* at late stages of priming, 120 h after bacterial inoculation (Figure 3A). Such transcript accumulation patterns are typical of the priming response. Inoculation of unprimed plants with PspNPS3121 had only a small effect on transcript accumulation compared to the response of the primed plants.

Likewise, INA treatment by itself did not induce transcription of *PvWRKY6, PvWRKY29, PvWRKY53, PvPR1, PvPR4*, and *PvNPR1* (Figure 3), in systemic leaves 24 hours after treatment, but caused considerable transcript accumulation for *PvWRKY29* and *PvWRKY53* genes 5 days after inoculation with the pathogen. Thus, common bean plants were primed for potentiated gene activation, which was subsequently induced by pathogen infiltration.

### Distinct chromatin modifications prime distinct defense genes for activation of expression

**Figure 4:**
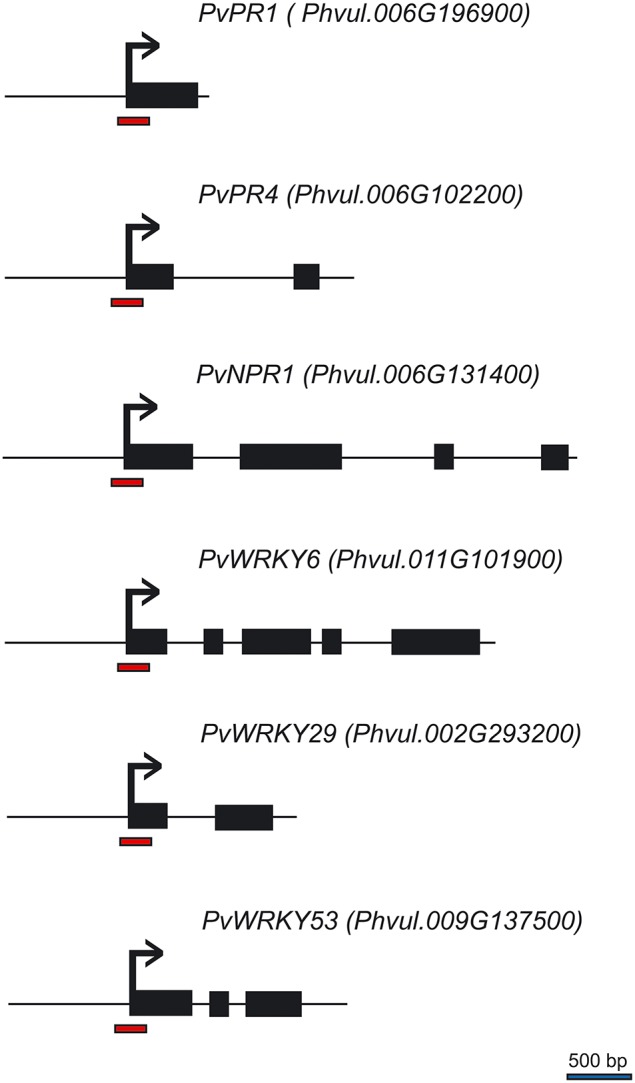
**Schematic representation of the *P. vulgaris* genes analyzed by ChIP**. Black boxes represent exons, horizontal lines represent introns, red bars at the promoter-exon boundary region show the segments amplified by PCR, and the bent arrows represent the transcription start site.

In the previous experiment, we observed that there was increased accumulation of transcripts for defense genes when plants were first primed with the synthetic activators before they were challenged with the pathogen. We asked if the increased transcript accumulation was associated with changes in chromatin structure at the promoter region of these genes. To address this, we performed chromatin immunoprecipitation (ChIP) analysis on their promoter DNA using antibodies against H3K4me3 and H3K36me3; this should provide information on the H3K4me3 and H3K36me3 status of the nucleosomes at the promoter-exon boundary region. The genes are schematically represented in Figure 4. Of particular interest was the status of the putative positioned nucleosome at the transcriptional start site, which is flanked up-stream by a promoter nucleosome-free region (Workman, 2006; Li et al., 2014; Zhang et al., 2015).

**Figure 5:**
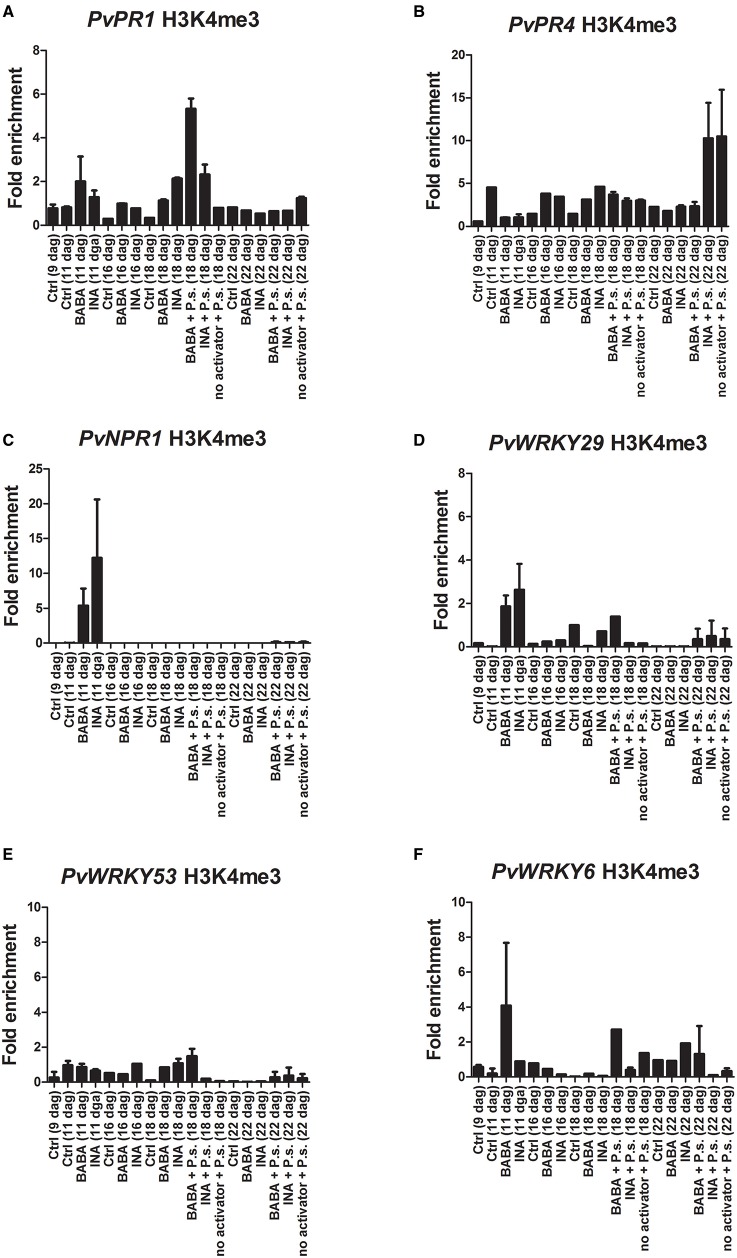
**Histone methylation profiles of the *P. vulgaris* genes involved in plant defense at various days after germination (dag)**. Plants were primed with activators and later inoculated with *P. syringae* pv. phaseolicola (Activator + P.s.), inoculated only (no activator + P.s.), or neither primed nor inoculated (control, ctrl). ChIP assays with antibodies specific for H3K4me3 in BABA-primed and INA-primed plants. **(A)** *PvPR1*; **(B)** *PvPR4*; **(C)** *PvNPR1*; **(D)** *PvWRKY29*; **(E)** *PvWRKY53*; **(F)** *PvWRKY6*. Depletion of H3K4me3 from the promoter-exon boundary region correlates with enhanced transcription of the primed genes. Two independent biological assays are shown.

ChIP analysis of the H3K4me3 and H3K36me3 chromatin marks at the promoter-exon boundary region showed that tri-methylation of H3K4 (H3K4me3) increased 24 h after BABA application on the promoter of *PvWRKY29* (Figure 5D) and *PvWRKY6* (Figure 5F), even though it was not accompanied by increased transcription of *PvWRKY6* and *PvWRKY29* (Figures 5D,F, respectively). There was no enrichment in H3K36me3 (Supplementary Figure 3). In addition, BABA application enhanced the H3K4me3 and H3K36me3 modifications 24 h after priming at the promoter of the *PvPR1* gene (Figure 5A and Supplementary Figure 3, respectively), although there was only minor transcript accumulation at this time (Figure 3). Whereas INA application enhanced the H3K36me3 mark of the *PvPR1* gene 24 h after priming (Supplementary Figure 3). Furthermore, BABA enhanced the H3K4me3 mark on the *PvNPR1* gene (Figure 5C), with only minor transcriptional activation of this gene (Figure 3). Twenty-four hours after application of INA, H3K4me3 was enriched at the promoter of *PvWRKY29* (Figure 5D) and H3K36me3 was enriched at the promoter of *PvWRKY53* (Supplementary Figure 3). However, in the promoter-exon boundary region of *PvNPR1*, INA stimulus enriched both H3K4me3 and H3K36me3 marks (Figure 5C and Supplementary Figure 3) without enhanced transcription (Figure 3). These results indicate that the different chromatin marks associated with active gene expression are induced during the 24 h after chemical priming, before actual activation of gene transcription, and are activator- and gene-specific.

Twenty four hours after PspNPS3121 inoculation there was a decrease in the H3K4me3 and H3K36me3 marks (with respect to the modifications 24 h after priming), while transcript levels were enhanced for *PvPR4, PvNPR1, PvWRKY29, PvWRKY53*, and *PvWRKY6* transcripts (mainly for BABA + P.s. treated plants; Figure 3). At this time, the chromatin marks that had been enriched by priming were restored to basal levels (Figure 5); that is, the gene-specific nucleosomes were absent from the H3K4me3- or H3K36me3-bound fraction and the immunoprecipitated DNA from the ChIP assay was no longer enriched for those particular marks. We conclude that, at this time, there was an inverse correlation between transcript accumulation and chromatin modification (Figure 3). This is the typical biphasic priming curve: enriched activating chromatin marks accompanied by basal levels of transcript accumulation during the priming phase and the reverse after the challenge: basal levels of chromatin marks and enhanced transcript accumulation.

Five days after inoculation, transcript levels were high mainly for *PvPR1* (BABA + P.s.; Figure 3A) *PvWRKY29* (INA + P.s.; Figure 3D), and *PvWRKY53* (INA + P.s.; Figure 3E) while their H3K4me3 and H3K36me3 marks were low at that stage. That is, basal levels of chromatin marks and enhanced transcript accumulation. This suggests a late expression of these genes for those particular treatments.

The loss of the chromatin marks after pathogen infection could be due to many factors. These include nucleosome eviction to promote transcription, nucleosome repositioning during transcription, histone H3 replacement, or because the H3K4me3 and H3K36me3 chromatin marks provide docking sites for chromatin remodeling factors or other regulatory proteins that would hinder antibody binding. Whatever the case, the different priming activator agents enriched the H3K4me3 and H3K36me3 chromatin marks and did not activate gene expression 24 h after their application. However, upon PspNPS3121 inoculation there was accumulation of transcripts and a decrease in the activating H3K4me3 and H3K36me3 chromatin marks at the promoter-exon boundary region of the different defense-related genes.

### Transgenerational inheritance of priming in the common bean

**Figure 6:**
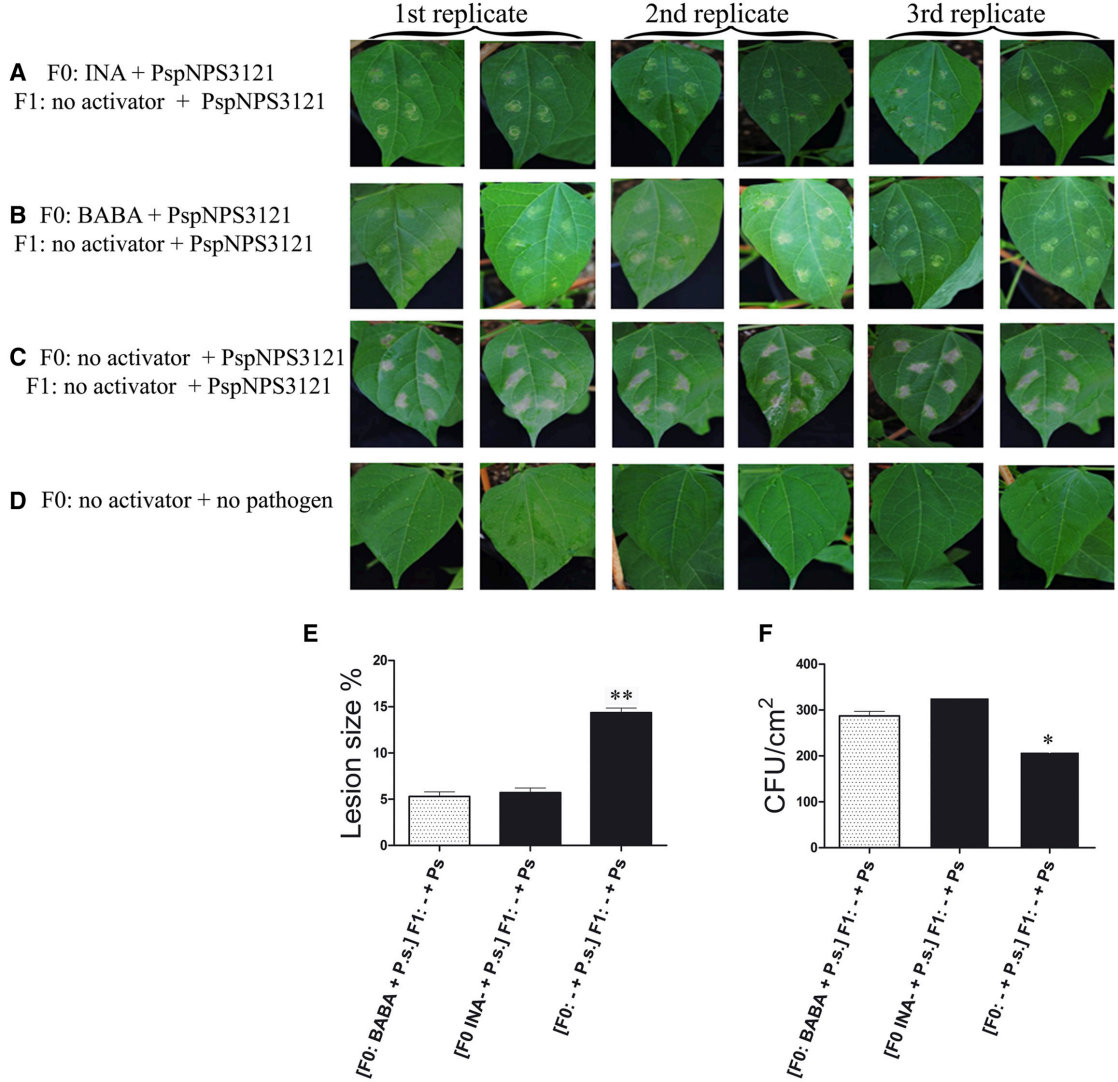
**Disease symptoms in unprimed, 2-week-old F1 progeny of *P. vulgaris* plants inoculated with *P. syringae* pv. phaseolicola (P.s.)**. F0 were: primed with activators and inoculated (Activator + P.s.), unprimed and inoculated (− + P.s), or neither primed nor inoculated. **(A–D)** photos of F1 leaves after inoculation; **(E)** Lesion size; and **(F)** CFUs. Data represent mean ± SD, *n* = 3 independent experiments; data were analyzed with an unpaired two-tailed Student's *t*-test (^*^p < 0.05, ^**^p < 0.01).

This study was undertaken to determine if primed *P. vulgaris* plants that had been exposed to pathogens would produce progeny that were more resistant to disease than progeny of unprimed parents, a phenomenon is referred to as transgenerational resistance. The F0 generation consisted of primed, inoculated plants; unprimed, inoculated plants; and unprimed, non-inoculated plants. The F1 generations were progeny from a self-cross of these parents. Two-week-old progeny from all crosses were challenged with PspNPS3121 (without priming) and assessed for disease symptoms, specifically lesion size and number of CFUs. The results are shown in Figure 6. Pathogen colonization was reduced in F1 progeny from parents that had been both primed and inoculated compared to the progeny from the other two groups of parents. Thus, F1 plants from parents that were primed and inoculated were more resistant. Furthermore, F1 plants that were only treated with the pathogen in the F0 and F1 generations, and were, therefore, under continuous stress had a hypersensitive response (HR). That is, the plants presented some type of early defense response that caused necrotic lesions around the infection site and cell death to restrict the growth of the pathogen (Figure 6C).

**Figure 7:**
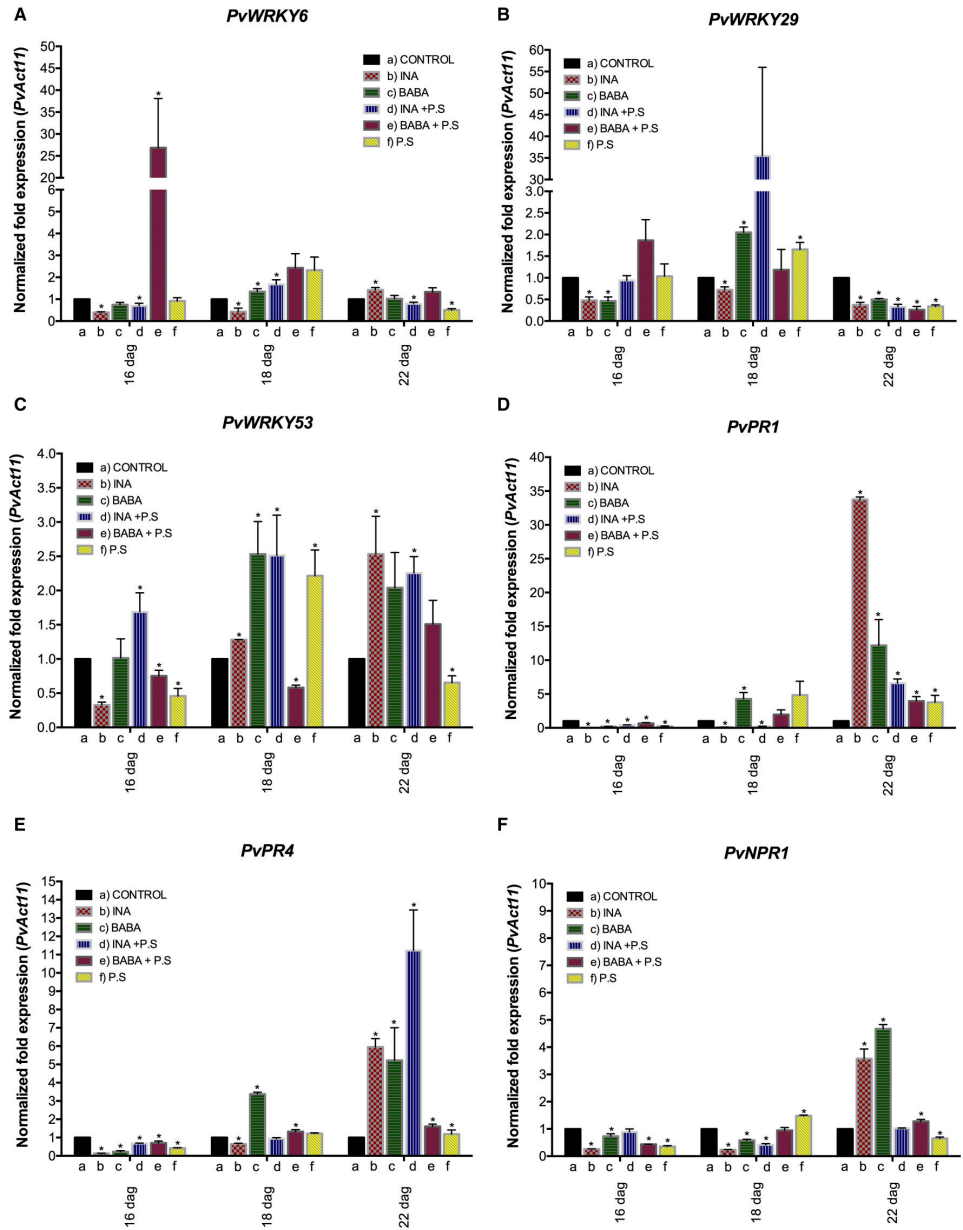
**Transcript levels in F1 progeny of selected genes involved in plant defense**. Progeny were either: unprimed and not inoculated (−) or unprimed and inoculated with *P. syringae* pv. phaseolicola (− + P.s.). F1 progeny were descended from F0 plants that had been primed with activator and inoculated with *P. syringae* pv. phaseolicola (Activator + P.s.), inoculated only (− + P.s.), or neither primed nor inoculated (Ctrl 1 or 2). Data were normalized to the Actin11 (*PvActin11*) reference gene. **(A)** *PvWRKY6*; **(B)** *PvWRKY29*; **(C)** *PvWRKY53*; **(D)** *PvPR1*; **(E)** *PvPR4*; **(F)** *PvNPR1*. Data represent mean ± SD, *n* = 3 independent experiments. Statistical significance for the F1 generation was determined with multiple Student's *t*-test, followed by the Holm-Šídák multiple comparison test at a significance value of 0.05 (^*^p < 0.05), by using the GraphPad Prism (v 6.0, GraphPad Software, San Diego California USA, www.graphpad.com). A one-way ANOVA with Dunnett's post-test was performed using GraphPad Prism (v 6.0, GraphPad Software, San Diego California, USA) at a significance value of 0.05 (see Supplementary Table 2).

Next, we wished to examine transcription of the candidate genes in the F1 generation. The F1 progeny were inoculated with PspNPS3121 and the transcript levels of *PvWRKY6, PvWRKY29, PvWRKY53, PvPR1, PvPR4*, and *PvNPR1* were assessed by q-PCR and compared to non-inoculated plants 5 days later. As shown in Figure 7 (and in Supplementary Figure 4), the only genes with inoculation-induced elevated transcript levels in the F1 from parents that had been primed and inoculated were *PvWRKY29* and *PvPR4* (Figures 7B,E). Furthermore, this pattern of gene expression occurred only when the parent plant had been primed with INA and not BABA. However, enhanced transcription accumulation for *PvWRKY29* occurred 24 h after bacterial inoculation (Figure 7B), whereas transcription accumulation for *PvPR4* took place 120 h after infection (Figure 7E). Consequently, we next asked if transcript accumulation is associated with changes in chromatin structure at the promoter region of these genes. We addressed this question with ChIP analysis of the F1.

**Figure 8:**
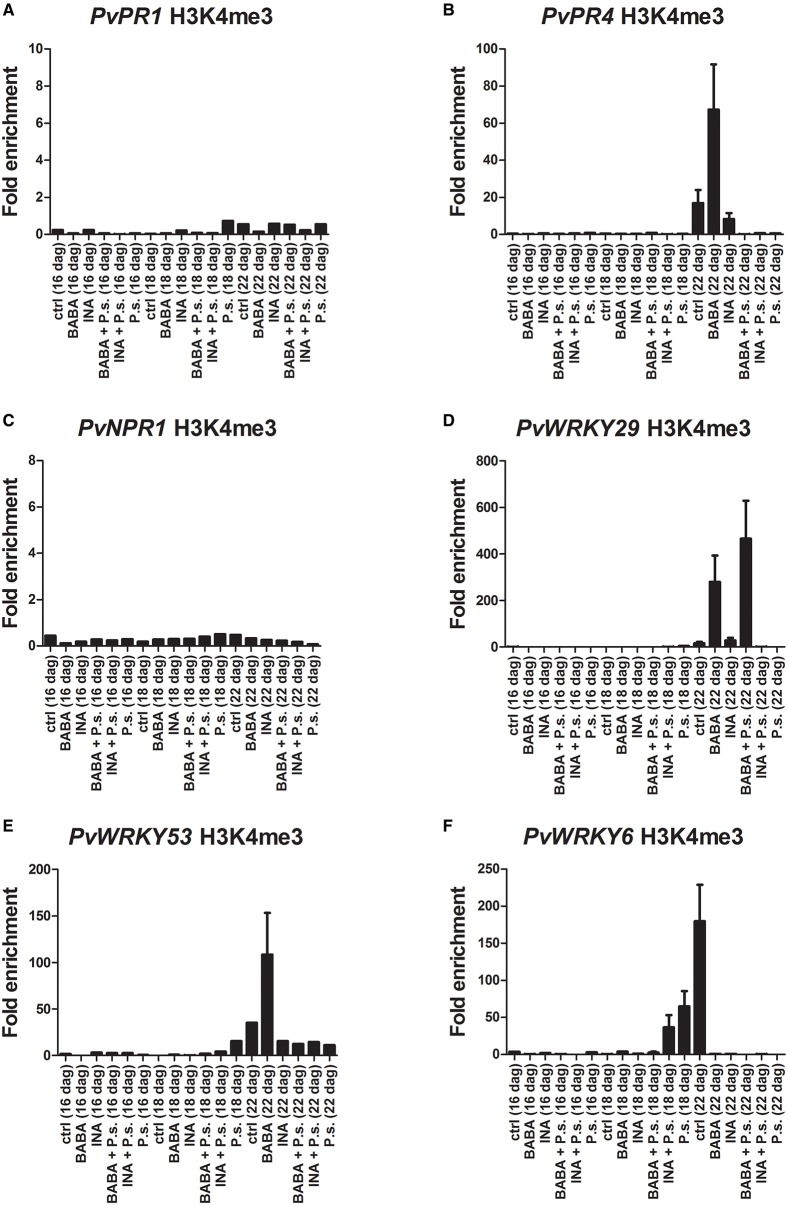
**Histone methylation profiles as determined by ChIP assays with antibodies specific for H3K4me3**. F1 progeny were descended from F0 plants that had been primed with activator and inoculated with *P. syringae* pv. phaseolicola (Activator + P.s.), inoculated only (− + P.s.), or neither primed nor inoculated (Ctrl). **(A)** *PvPR1*; **(B)** *PvPR4*; **(C)** *PvNPR1*; **(D)** *PvWRKY29*; **(E)** *PvWRKY53*; **(F)** *PvWRKY6*. Two independent biological assays are shown.

The results of the ChIP analysis of the F1 progeny are shown in Figure 8 (and in Supplementary Figure 5). Twenty four hours before inoculation and 24 h after inoculation with PspNPS3121 the F1 plants were not enriched in the H3K4me3 mark at the promoter region of the different gene. These plants also had little transcript accumulation at those stages (Figure 7). However, 5 days after they had been inoculated, transcript levels were increased for *PvPR1, PvPR4*, and *PvNPR1* and the H3K4me3 mark at the promoter of these genes decreased (Figure 8).

On the contrary, transcript levels were lower (5 days after inoculation), for *PvWRKY29* and *PvWRKY6*, and the H3K4me3 and H3K36 marks at their promoters was increased. This phenomenon was mainly seen in progeny from INA-treated plus pathogen-inoculated plants.

## Discussion

As an essential food and feed crop worldwide, there are ongoing, major efforts to improve the common bean. These efforts, at present, are mainly limited to conventional breeding practices, such as the development of cultivars with improved disease resistance, as this crop is difficult to regenerate *in vitro*. A promising alternate approach is epigenetics, which may have the potential to enhance the heritable natural variation that exists. Stable epigenetic variants that affect plant traits have been identified and indicate that epigenetic variation is a component of natural phenotypic variation (Springer, 2013). Indeed, it is likely that some of the quantitative trait loci that have been utilized by breeders are due to epigenetic, rather than genetic, variation.

At present, defense priming is regarded as a fundamental process in various types of systemic plant immunity. Defense-priming processes include: SAR, induced systemic resistance (ISR), resistance conferred by arbuscular mycorrhizal fungi, β-aminobutyric acid-induced immunity (BABA-IR), and wound-induced resistance (Conrath et al., 2015). Although the molecular mechanisms of defense priming were unclear for years, it is now considered that chromatin modifications prime defense genes for faster and stronger transcription and that such mechanisms enable long-term stress imprints to be left in the plant (Bruce et al., 2007).

For example, DNA can be methylated, whereas the N-termini of histones can be acetylated, methylated, phosphorylated, or ubiquitinated. It is currently recognized that acetylation of histone lysine residues activate gene transcription (Eberharter and Becker, 2002). Also, histone lysine methylation patterns are associated with activation and repression of gene transcription. Thus, it has been suggested that histone modifications could take place at the onset of priming (van den Burg and Takken, 2009). Furthermore, apparently localized bacterial infection initiates distribution of systemic signal(s) that are converted and stored as modifications to histones in defense gene promoters in systemic leaves. Consequently, histone modifications seem to provide a within-generation memory for priming in the systemic plant immune response (Jaskiewicz et al., 2011; Conrath et al., 2015).

Epigenetic changes relating to DNA modification display greater heritability. Transgenerational phenotypic changes appear either by gain or loss of methylations at cytosines (Kumar et al., 2015). They are inherited through cell divisions and can be transmitted to the next generation. They therefore offer a possible mechanism for stress memories in plants (Kinoshita and Seki, 2014). However, as the genome of many organisms with transgenerational memory (e.g., flies, worms) does not encode DNA methyltransferases, next-generation priming cannot provide a universal epigenetic memory; it may be limited to plants (Conrath et al., 2015). Accordingly, two recently published papers report the inheritance of ectopically induced domains of the histone modification H3K9me through numerous mitotic and meiotic cell divisions in the absence of DNA sequence-specific initiator, in the fission yeast (Audergon et al., 2015; Ragunathan et al., 2015). Such results show that, thus far, in *Schizosaccharomyces pombe* histones can act as carriers of epigenetic information independently of the underlying DNA sequence (Jones, 2015).

Thus, the goals of this work were: first, to activate a primed state of enhanced defense in the common bean against PspNPS3121 through the application of effective synthetic priming activators. Second, we undertook to identify the chromatin state required for inducible defense against pathogens. We hoped that successful accomplishment of these goals would allow us to analyze the epigenetic processes involved in the interaction between plants and microorganisms, and serve to improve future plant breeding and crop productivity. To this end, we studied the effect of priming with INA and BABA on gene activation and the inheritance of the primed state.

### Generational priming

Application of the priming activators INA or BABA at the moderate concentration of 100 μM (Sticher et al., 1997; Ton and Mauch-Mani, 2004; Arasimowicz-Jelonek et al., 2013) effectively primed and protected common bean plants against infection by PspNPS3121 in this study. There was a reduction of lesion size and CFU in treated plants, compared with the untreated control. Next, to identify genes of defense priming that are candidates for markers of priming, we analyzed the transcript levels of *PvWRKY6, PvWRKY29, PvWRKY53, PvPR1, PvPR4*, and *PvNPR1* before and after INA and BABA application, as well as before and after inoculation with *P. syringae* pv. phaseolicola. The plant *WRKY* genes code for transcription factors (TFs) involved in the regulation of developmental processes. They are induced in response to different stresses and act to increase stress tolerance (Agarwal et al., 2011). In *Arabidopsis*, for example, transcripts of *WRKY* are increased in systemic tissues after SAR-induction with biological or chemical agents and increased further after secondary pathogen inoculation (Jaskiewicz et al., 2011; Singh V. et al., 2014). The *Arabidopsis PR1* and *NPR1* genes are involved in transgenerational SAR (Kohler et al., 2002; Luna et al., 2012) and the Arabidopsis *PR4* gene is induced by biotic stresses, activators of SAR, and wounding (Thomma et al., 2001; Bertini et al., 2003, 2012).

All the genes analyzed in this study showed a typical priming response with respect to their transcription patterns, that is, application of the priming agents did not activate gene expression, but rather, led to a stronger and faster activation of defense genes upon attack by the pathogen PspNPS3121. BABA primed the six genes analyzed and INA primed only two genes, as compared to unprimed, but stressed, plants. This indicates that the extent of the priming response is dependent on the priming agent used, as well as on the time after pathogen infection. Thus, the enhanced resistance conferred by BABA correlated with primed transcription of *PvWRKY6, PvWRKY29, PvWRKY53, PvPR1, PvPR4*, and *PvNPR1* genes (24 h after inoculation, except for *PvPR1* which showed an enhanced expression 120 h after pathogen inoculation); the enhanced resistance conferred by INA correlated with primed transcription of *PvWRKY29* and *PvWRKY53* genes.

The expression patterns shown here are similar to those reported for *Arabidopsis*. Zimmerli and colleagues found that BABA-induced resistance against *P. syringae* DC3000 in *Arabidopsis* correlated with primed transcription of the *PR1* gene (Zimmerli et al., 2000; Slaughter et al., 2012). Transcriptional priming of *Arabidopsis PR1, WRKY6*, and *WRKY53*, (as well as *PR5, WRKY70*, and *WRKY38*) was also observed in BABA-treated plants (Luna et al., 2014) and SAR-induced priming of *Arabidopsis WRKY6* and *WRKY29* has been demonstrated (Singh V. et al., 2014). Furthermore, INA induced *PR1* gene expression in tobacco (*Nicotiana tabacum*), accompanied by enhanced resistance of the treated plants to tobacco mosaic virus (Conrath et al., 1995).

The robust priming of gene expression that we observed encouraged us to use ChIP to examine histone methylation in the promoter-exon boundary region, as this might account for the priming effect. With the exception of *PvPR4*, we observed changes in histone methylation, after priming, in all of the genes we studied. Specifically, the promoters of the different genes had elevated levels of H3K4me3 and H3K36me3, a histone modification that correlates with their transcriptional capacity. Other workers have shown that these post-translational histone modifications are associated with defense-gene priming by synthetic activators. For example, priming of the promoter of the *Arabidopsis WRKY6, WRKY29*, and *WRKY53* genes by BTH treatment has been associated with H3K4me3, H3K4me2, and acetylation of histone H3 and H4 (Jaskiewicz et al., 2011). More recently, Singh and colleagues showed that *Arabidopsis* plants exposed to recurrent heat, cold, or salt stress were more resistant to virulent bacteria and had enriched H3K4me3, H3K4me2, and acetylation of histone H3 at Lys-9 and Lys-14 (H3K9K14ac) in the promoter region of the *Arabidopsis* genes *FRK1, WRKY53, NHL10, WRKY70*, and *PR1* genes, than unstressed plants (Singh P. et al., 2014).

H3K4me3 increased 24 h after BABA application on the *PvWRKY6* promoter-exon boundary region, in spite of the fact that *PvWRKY6* transcription was not induced at that time. Similar patterns of enriched H3K4me3 without transcript accumulation were observed at the promoter-exon boundary regions of *PvWRKY29, PvPR1*, and *PvNPR1* after BABA treatment and at the promoters of *PvWRKY29* and *PvNPR1* after INA treatment. Also, enriched H3K36me3 was present in the same region for *PvPR1* and *PvNPR1* after BABA treatment and in *PvWRKY53* and *PvNPR1* after INA application. Thus, these particular chromatin marks, which enhance gene expression, are induced in the common bean during chemical priming before actual activation of gene transcription and may facilitate gene transcription upon future challenges (Conrath et al., 2015).

Perhaps predictable from this observation, we observed a faster and stronger activation of these genes upon inoculation with the pathogen PspNPS3121. It is also noteworthy that, upon inoculation accompanied by activation of gene expression, the H3K4me3 and H3K36me3 chromatin marks were either not detected after the immunoprecipitation or disappeared from the promoter-exon boundary region. This may be because the H3K4me3 and H3K36me3 chromatin marks provide docking sites in active promoters for transcriptional co-activators, chromatin remodeling factors, or other regulatory proteins, which could hinder antibody binding (de la Cruz et al., 2005; Vermeulen et al., 2007). This might also be due to nucleosome eviction during recruitment of the components of the transcription pre-initiation complex or the general transcription machinery to facilitate transcription initiation and gene transcription. It might also be due to nucleosome repositioning during transcription initiation and gene transcription (Workman, 2006), or to histone H3 replacement during gene transcription (for example, histone H3.3; Stroud et al., 2012). Whatever the reason, we suggest that these H3K4me3 and H3K36me3 chromatin marks act as a signal integration and storage event in the primed plant stress response (Conrath et al., 2015).

### Transgenerational priming

We examined transgenerational inheritance of priming and induced resistance against PspNPS3121 in this study. Primed common beans served as the F0 with appropriate unprimed controls. The F1 generation from self-crosses was analyzed for disease resistance and other evidence of inherited priming. Unprimed F1 plants from primed parents challenged with PspNPS3121 showed an enhanced basal level of disease resistance and a higher capacity to react to additional pathogen stress compared to the F1 from unprimed parents. However, *PvWRKY29* and *PvPR4* were the only genes with strongly enhanced expression in the progeny of the INA-chemically primed plants. The patterns of transcript accumulation were entirely different for each gene. While enhanced transcript accumulation for *PvWRKY29* occurred 24 h after bacterial inoculation, transcription accumulation for *PvPR4* took place 120 h after infection.

There was a persistent transgenerational state of priming in the form of constitutively enhanced *PvWRKY6* transcript accumulation in F1 progeny from parents that had been both inoculated and primed with BABA. However, after pathogen inoculation the transcripts levels for *PvWRKY6* returned to its basal level. In contrast, enhanced *PvPR1* expression was enhanced only in F1 plants that had been inoculated and that had parents that had been treated with INA only.

Interestingly, F1 progeny from INA-treated and inoculated parents were primed effectively, over just one generation, for enhanced activation of *PvWRKY29* and *PvPR4* after pathogen inoculation only. That is, these genes clearly showed the typical priming response of increased transcription in the F1 progeny only after pathogen inoculation. *PvWRKY29* had a histone methylation pattern at the promoter-exon boundary region that could explain the priming behavior, while these plants had an associated resistance to PspNPS3121. Disappearance of H3K4me3 from the transcription start site after inoculation correlates with enhanced transcription activation of the presumably primed gene.

Thus, for these genes, the distinct primed states with respect to enhanced transcription were maintained over one generation and transferred to the descendants generated through mitotic divisions. Furthermore, from the results presented here, it appears to be a connection for the *PvWRKY29* gene between H3K4me3 as a molecular footprint to gene priming as the functional outcome (Jaskiewicz et al., 2011). Our results also suggest that the molecular mechanisms of defense-priming inheritance may rely on the priming agent to whom parental lines were exposed, the time after pathogen infection, as well as on the type of stress.

Consequently, for transgenerational inheritance of priming, the parents (F0) have to be able to recognize the specific stress, store this information, perhaps in the form of histone or DNA modifications, and pass it on to the progeny (Slaughter et al., 2012). To take advantage of this situation, the progeny has to be able to retrieve the information and use it for enhanced resistance against abiotic or biotic stress, as we have demonstrated here.

## Conclusions

The results presented here show that the treatment of common bean plants with INA or BABA induces a primed state that is passed on to the progeny. The progeny of primed plants showed an enhanced basal level of disease resistance and a higher capacity to react to additional biotic stress. We believe that the generational and transgenerational regulation of the *PvWRKY29* gene (and to a lesser extent the *PvPR4* gene), could be caused, in part, by the histone methylation status at the promoter-exon boundary region of this gene. These results could provide valuable knowledge for producing disease-resistant crop varieties by exposing parental plants to priming activators.

This study is important as it should broaden understanding of the epigenetic mechanisms involved in the interaction of plants and microorganisms and increase our knowledge of the epigenetic components of stress signaling and priming. Understanding of epigenetically controlled defense priming should be of value in sustainable agriculture.

## Author contributions

RA provided the idea of the work. RA and KM designed the experiments. KM and GR, conducted the phenotypic analysis, RT-PCR, qRT-PCR, and ChIP assays. JH performed RT-PCR, and ChIP assays. AB performed the statistical analysis. RA, KM, and GR participated in the interpretation of results and critically reviewed the manuscript. RA wrote the paper. All authors read and approved the final manuscript.

### Conflict of interest statement

The authors declare that the research was conducted in the absence of any commercial or financial relationships that could be construed as a potential conflict of interest.

## Supplementary material

Supplementary Figure 1 **Transcript levels of selected genes involved in plant defense in *P. vulgaris* plants as determined by end-point PCR**. Plants were primed with activators (BABA- or INA-treated plants) followed by inoculation with *P. syringae* pv. phaseolicola (Activator + P.s.), inoculated only (no activator + P.s.), or neither primed nor inoculated (control, ctrl). Data represents the densitometric analysis of defense-related gene expression. *PvActin11* was used as a control for each template preparation and was amplified under exactly the same conditions as the tested genes. Dag, days after germination.

Supplementary Figure 2 **Transcript levels of genes from *P. vulgaris* involved in plant defense as determined by qRT-PCR at various days after germination (dag)**. Plants were primed with activators (BABA- or INA-treated plants) followed by inoculation with *P. syringae* pv. phaseolicola (Activator + P.s.), inoculated only (no activator + P.s.), or neither primed nor inoculated (control, ctrl). Data were normalized to the elongation factor 1-α (*PvEF1*α) reference gene. Data represent mean ± *SD, n* = 3 independent experiments. Statistical significance for the F0 generation was determined with multiple Student's *t*-test, followed by the Holm-Šídák multiple comparison test at a significance value of 0.05, by using the GraphPad Prism (v 6.0, GraphPad Software, San Diego California USA, http://www.graphpad.com) (see Supplementary Table 1).

Supplementary Figure 3 **Histone methylation profiles of the *P. vulgaris* genes involved in plant defense at various days after germination (dag)**. Plants were primed with activators and later inoculated with *P. syringae* pv. phaseolicola (Activator + P.s.), inoculated only (no activator + P.s.), or neither primed nor inoculated (control, ctrl). ChIP assays with antibodies specific for H3K36me3 in BABA-primed and INA-primed plants. Depletion of H3K36me3 from the promoter-exon boundary region correlates with enhanced transcription of the primed genes. Two independent biological assays are shown.

Supplementary Figure 4 **Transcript levels in F1 progeny of selected genes involved in plant defense**. Progeny were either: unprimed and not inoculated (−) or unprimed and inoculated with *P. syringae* pv. phaseolicola (− + P.s.). F1 progeny were descended from F0 plants that had been primed with activator and inoculated with *P. syringae* pv. phaseolicola (Activator + P.s.), inoculated only (− + P.s.), or neither primed nor inoculated (Ctrl 1 or 2). Data were normalized to the elongation factor 1-α (*PvEF1*α) reference gene. Data represent mean ± SD, *n* = 3 independent experiments. Statistical significance for the F1 generation was determined with multiple Student's *t*-test, followed by the Holm-Šídák multiple comparison test at a significance value of 0.05, by using the GraphPad Prism (v 6.0, GraphPad Software, San Diego California USA, http://www.graphpad.com). A one-way ANOVA with Dunnett's post-test was performed using GraphPad Prism (v 6.0, GraphPad Software, San Diego California USA) at a significance value of 0.05 (see Supplementary Table 2).

Supplementary Figure 5 **Histone methylation profiles as determined by ChIP assays with antibodies specific for H3K36me3**. F1 progeny were descended from F0 plants that had been primed with activator and inoculated with *P. syringae* pv. phaseolicola (Activator + P.s.), inoculated only (− + P.s.), or neither primed nor inoculated (Ctrl). Two independent biological assays are shown.

Supplementary Table 1 **Multiple Student's t-test analysis, followed by the Holm-Šídák multiple comparison test at a significance value of 0.05**. Statistically significant values are in bold letters.

Supplementary Table 2 **One-way ANOVA with Dunnett's post test at a significance value of 0.05**. Statistically significant values are in bold.
