# Supplementary figures and images for: Use of BABA and INA As Activators of a Primed State in the Common Bean (Phaseolus vulgaris L.)
Source: Front Plant Sci. 2016 May 18;7:653. doi: 10.3389/fpls.2016.00653 (PMC4870254; doi:10.3389/fpls.2016.00653)

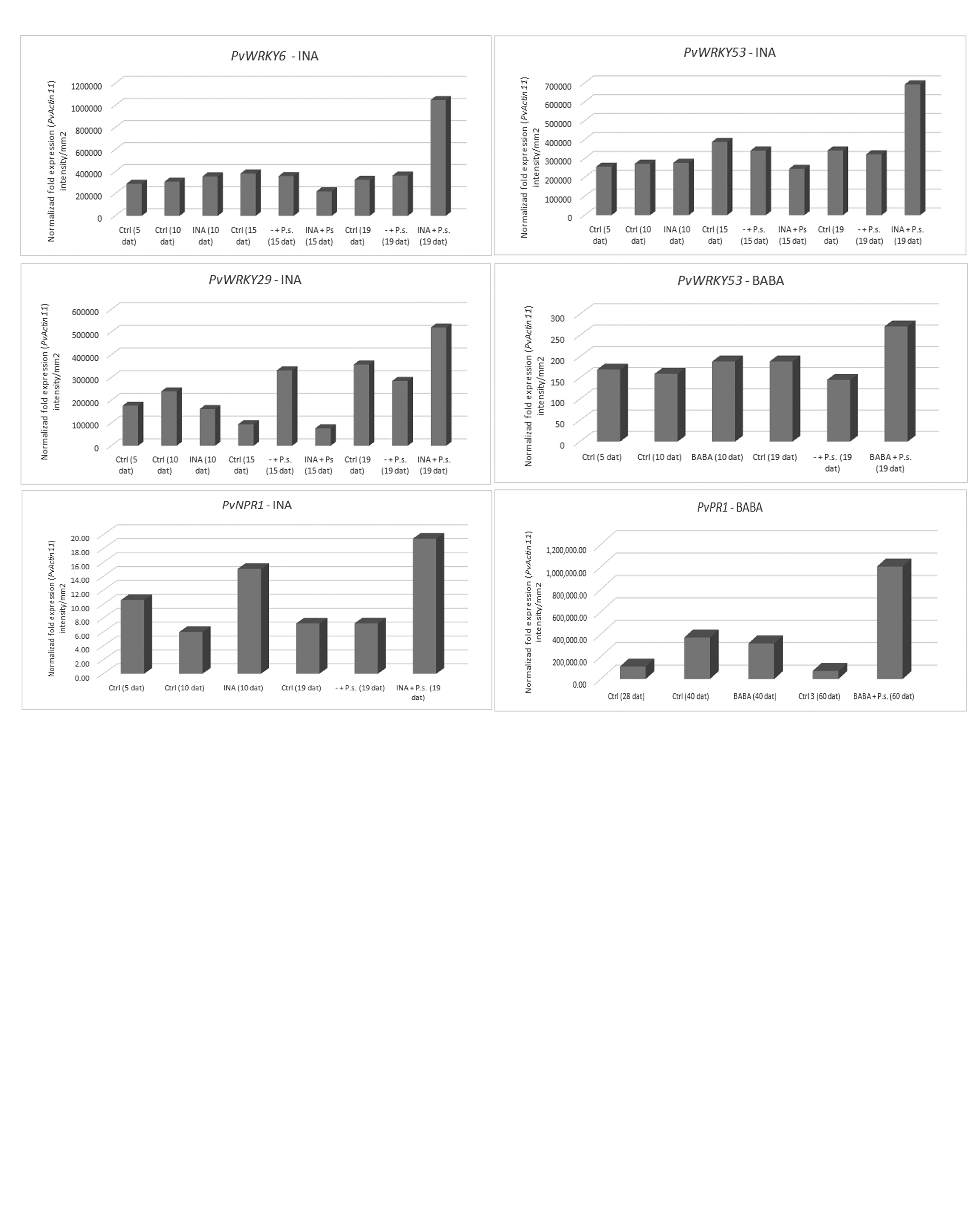

Supplement: Supplementary Figure 1 — Transcript levels of selected genes involved in plant defense in P. vulgaris plants as determined by end-point PCR. Plants were primed with activators (BABA- or INA-treated plants) followed by inoculation with P. syringae pv. phaseolicola (Activator + P.s.), inoculated only (no activator + P.s.), or neither primed nor inoculated (control, ctrl). Data represents the densitometric analysis of defense-related gene expression. PvActin11 was used as a control for each template preparation and was amplified under exactly the same conditions as the tested genes. Dag, days after germination. [file Image1.TIF]

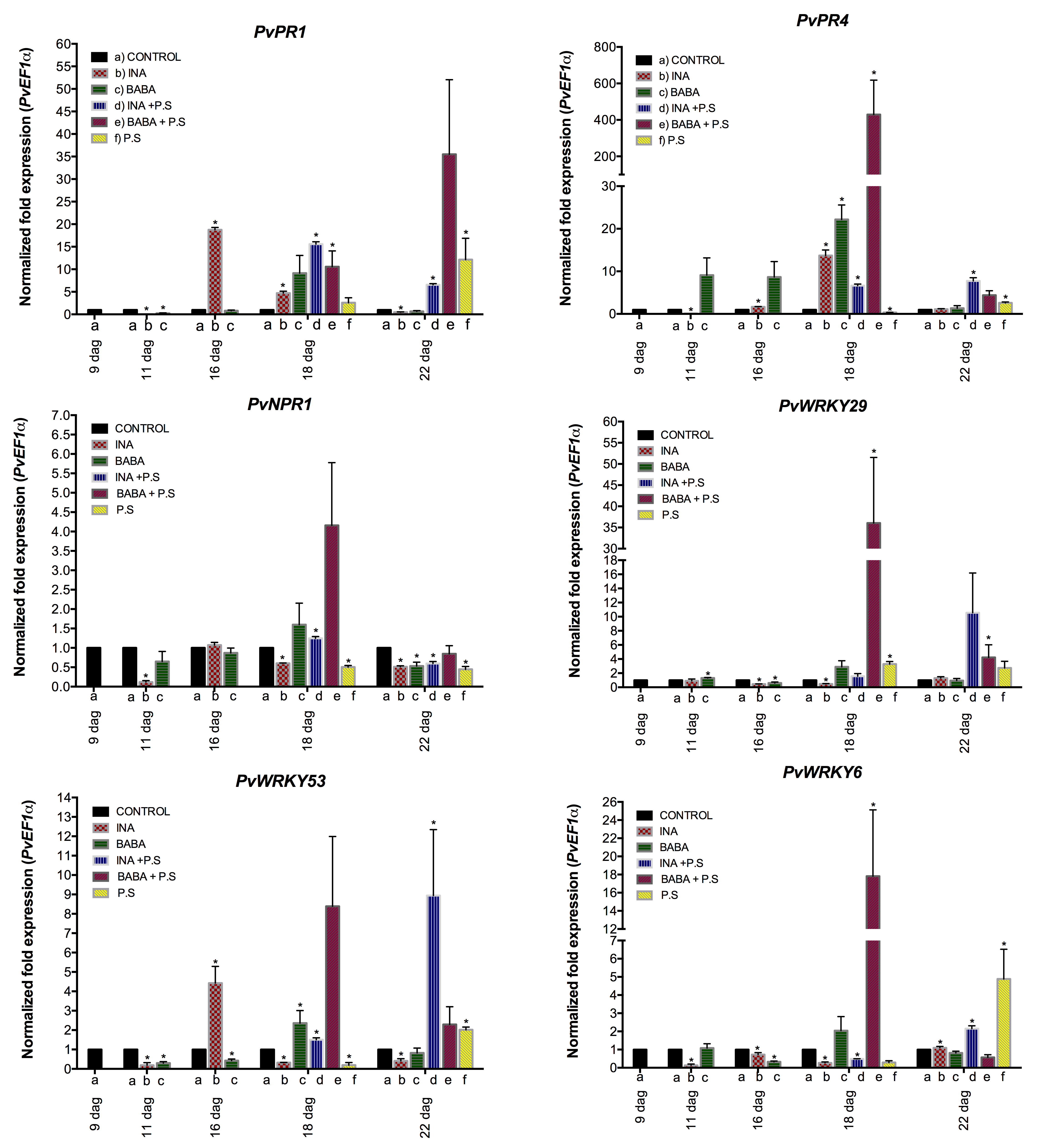

Supplement: Supplementary Figure 2 — Transcript levels of genes from P. vulgaris involved in plant defense as determined by qRT-PCR at various days after germination (dag). Plants were primed with activators (BABA- or INA-treated plants) followed by inoculation with P. syringae pv. phaseolicola (Activator + P.s.), inoculated only (no activator + P.s.), or neither primed nor inoculated (control, ctrl). Data were normalized to the elongation factor 1-α (PvEF1α) reference gene. Data represent mean ± SD, n = 3 independent experiments. Statistical significance for the F0 generation was determined with multiple Student's t-test, followed by the Holm-Šídák multiple comparison test at a significance value of 0.05, by using the GraphPad Prism (v 6.0, GraphPad Software, San Diego California USA, http://www.graphpad.com) (see Supplementary Table 1). [file Image2.TIF]

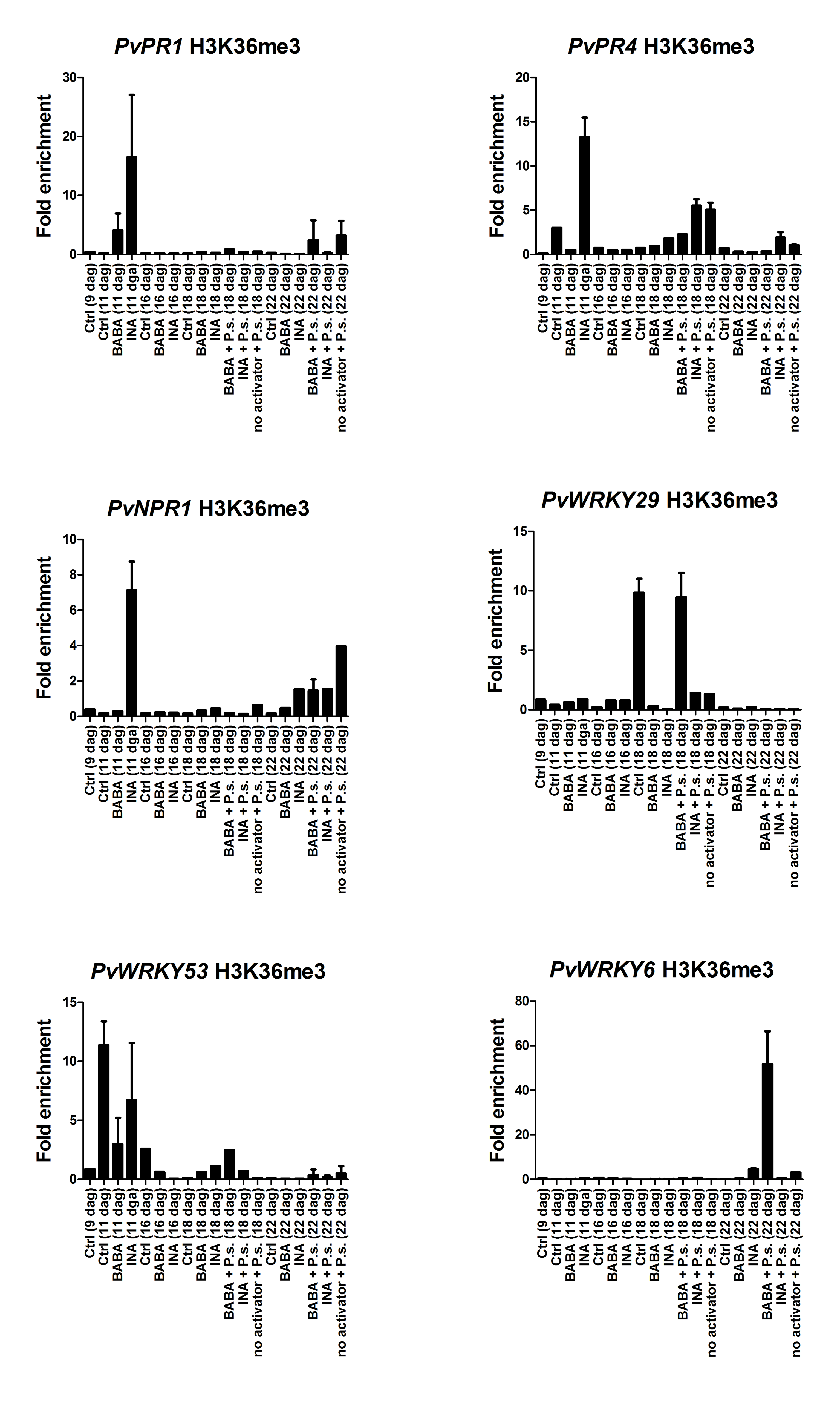

Supplement: Supplementary Figure 3 — Histone methylation profiles of the P. vulgaris genes involved in plant defense at various days after germination (dag). Plants were primed with activators and later inoculated with P. syringae pv. phaseolicola (Activator + P.s.), inoculated only (no activator + P.s.), or neither primed nor inoculated (control, ctrl). ChIP assays with antibodies specific for H3K36me3 in BABA-primed and INA-primed plants. Depletion of H3K36me3 from the promoter-exon boundary region correlates with enhanced transcription of the primed genes. Two independent biological assays are shown. [file Image3.TIF]

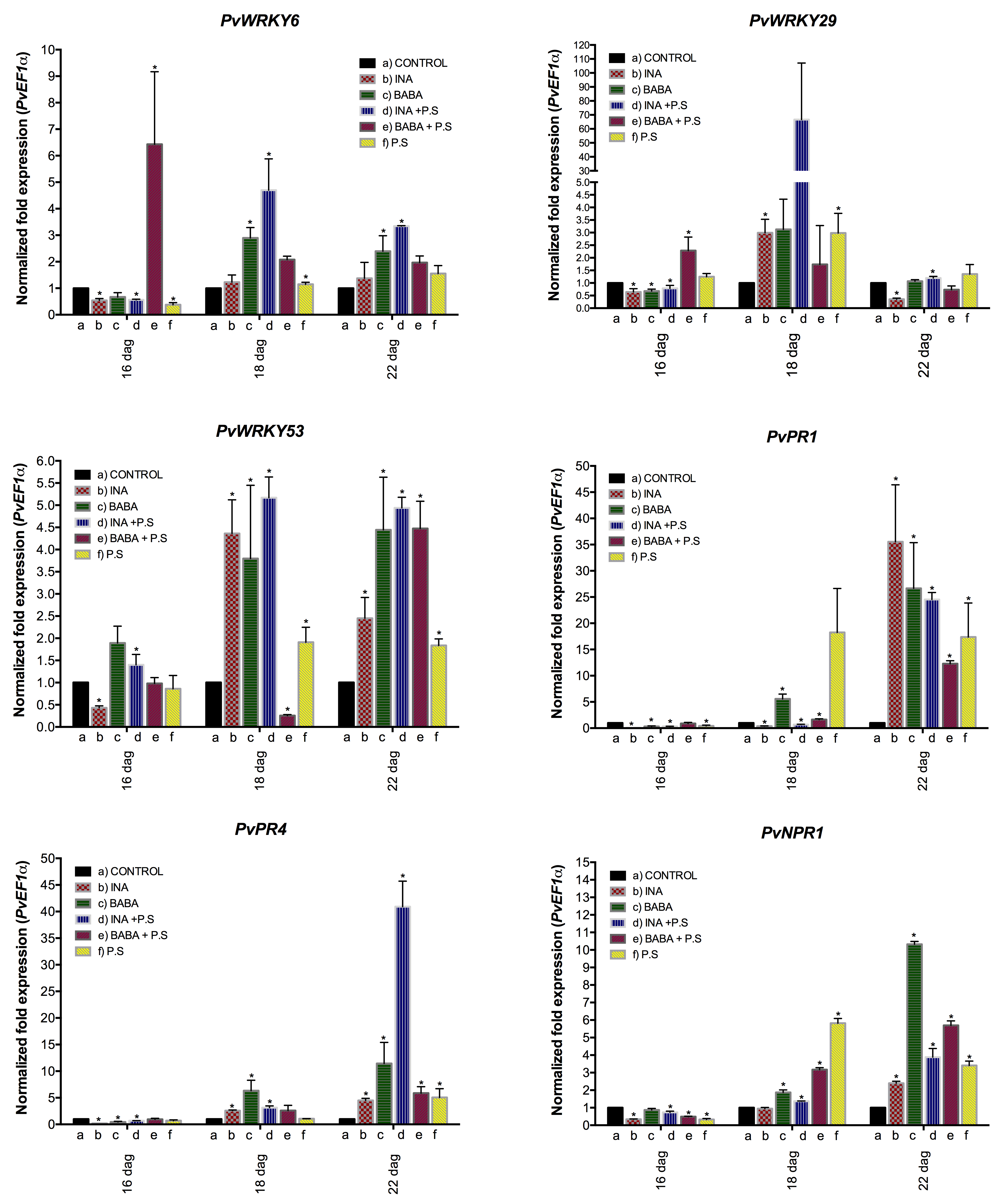

Supplement: Supplementary Figure 4 — Transcript levels in F1 progeny of selected genes involved in plant defense. Progeny were either: unprimed and not inoculated (−) or unprimed and inoculated with P. syringae pv. phaseolicola (− + P.s.). F1 progeny were descended from F0 plants that had been primed with activator and inoculated with P. syringae pv. phaseolicola (Activator + P.s.), inoculated only (− + P.s.), or neither primed nor inoculated (Ctrl 1 or 2). Data were normalized to the elongation factor 1-α (PvEF1α) reference gene. Data represent mean ± SD, n = 3 independent experiments. Statistical significance for the F1 generation was determined with multiple Student's t-test, followed by the Holm-Šídák multiple comparison test at a significance value of 0.05, by using the GraphPad Prism (v 6.0, GraphPad Software, San Diego California USA, http://www.graphpad.com). A one-way ANOVA with Dunnett's post-test was performed using GraphPad Prism (v 6.0, GraphPad Software, San Diego California USA) at a significance value of 0.05 (see Supplementary Table 2). [file Image4.TIF]

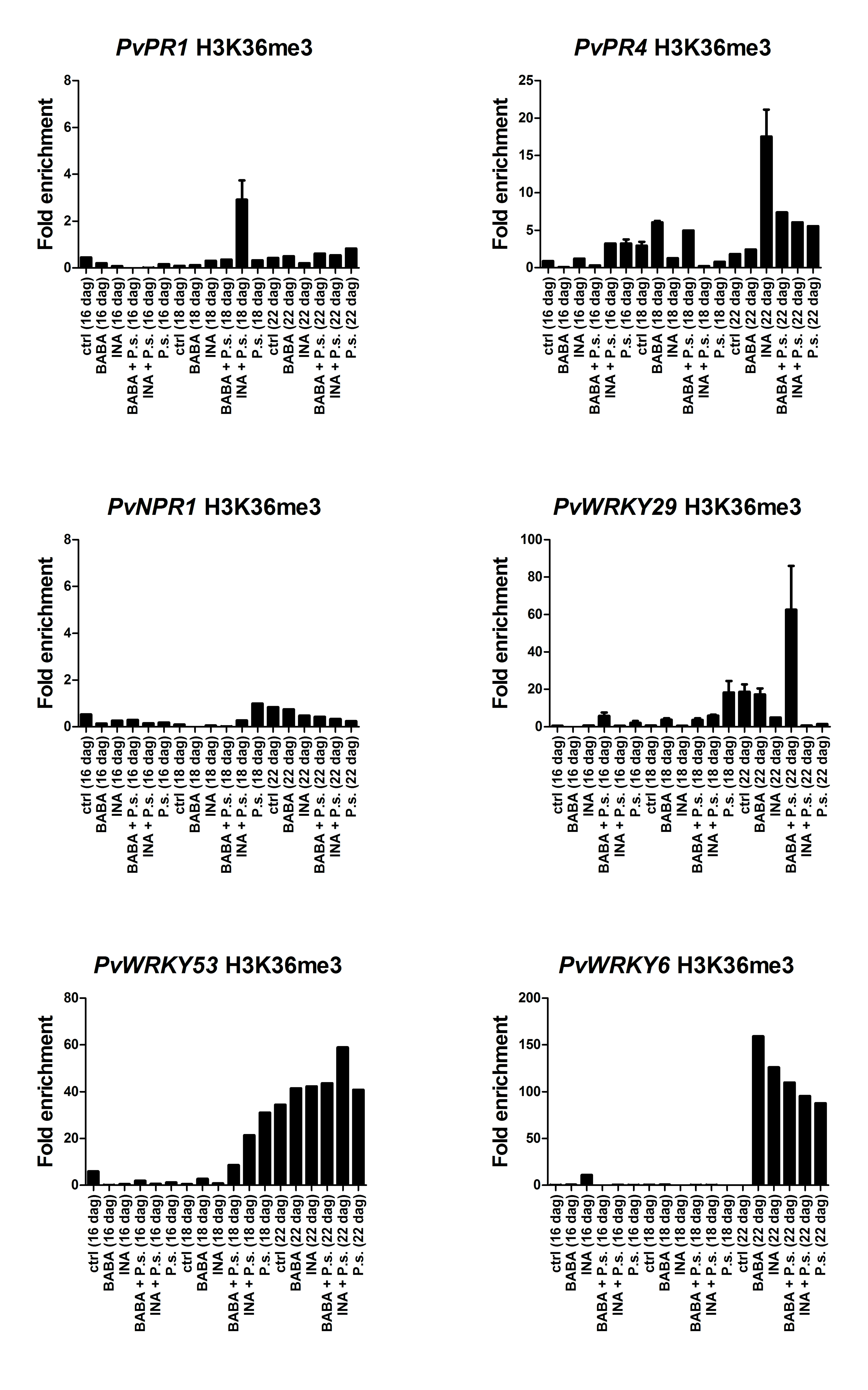

Supplement: Supplementary Figure 5 — Histone methylation profiles as determined by ChIP assays with antibodies specific for H3K36me3. F1 progeny were descended from F0 plants that had been primed with activator and inoculated with P. syringae pv. phaseolicola (Activator + P.s.), inoculated only (− + P.s.), or neither primed nor inoculated (Ctrl). Two independent biological assays are shown. [file Image5.TIF]
